# Tooth resorptions are not hereditary

**DOI:** 10.1590/2177-6709.22.4.022-027.oin

**Published:** 2017

**Authors:** Alberto Consolaro, Débora de Almeida Bianco

**Affiliations:** 1 Bauru School of Dentistry, Universidade de São Paulo (Bauru/SP, Brazil). Ribeirão Preto School of Dentistry, Universidade de São Paulo (Ribeirão Preto/SP, Brazil).; 2 School of Dentistry, Universidade Estadual de Maringá (Maringá/PR, Brazil).

**Keywords:** Tooth resorptions, Root resorptions, Tooth movement, Heredity.

## Abstract

Root resorptions caused by orthodontic movement are not supported by consistent scientific evidence that correlate them with heredity, individual predisposition and genetic or familial susceptibility. Current studies are undermined by methodological and interpretative errors, especially regarding the diagnosis and measurements of root resorption from orthopantomographs and cephalograms. Samples are heterogeneous insofar as they comprise different clinical operators, varied types of planning, and in insufficient number, in view of the prevalence of tooth resorptions in the population. Nearly all biological events are coded and managed through genes, but this does not mean tooth resorptions are inherited, which can be demonstrated in heredograms and other methods of family studies. In orthodontic root resorption, one cannot possibly determine percentages of how much would be due to heredity or genetics, environmental factors and unknown factors. There is no need to lay the blame of tooth resorptions on events taking place outside the orthodontic realm since in the vast majority of cases, resorptions are not iatrogenic. In orthodontic practice, when all teeth are analyzed and planned using periapical radiography or computerized tomography, and when considering all predictive factors, tooth resorptions are not iatrogenic in nature and should be considered as one of the clinical events inherent in the treatment applied.

## RESUMO

As reabsorções radiculares decorrentes da movimentação ortodôntica não têm evidência científica consistente que as correlacione com a hereditariedade, predisposição e suscetibilidade genética ou familiar. Os trabalhos sobre esse tema apresentam erros metodológicos e interpretativos, em especial quanto ao diagnóstico e à mensuração das reabsorções radiculares a partir de ortopantomografias e cefalogramas. As amostras são heterogêneas - quanto aos operadores clínicos e tipos de planejamentos aplicados - e em número muito pequeno, considerando-se a prevalência das reabsorções dentárias na população. Quase todos os eventos biológicos são codificados e gerenciados a partir dos genes, mas não por isso as reabsorções dentárias são hereditárias, o que seria demonstrado em heredogramas e outras formas de estudos familiares. Nas reabsorções radiculares em Ortodontia, não é possível determinar percentuais de quanto seria decorrente da hereditariedade ou da genética, de fatores ambientais e de fatores desconhecidos. Não se faz necessário transferir a “culpa” das reabsorções dentárias para eventos externos à Ortodontia pois, na grande maioria dos casos, elas não são iatrogênicas. Na prática ortodôntica, quando se faz a análise de todos os dentes e o planejamento, via radiografia periapical ou tomografia computadorizada, e quando se leva em consideração os fatores preditivos, as reabsorções dentárias não serão de natureza iatrogênica, e devem ser encaradas como uma das intercorrências clínicas do tratamento aplicado.

Tooth resorptions have several well-known causes, all with clearly established and substantiated mechanisms.^6^ A comprehensive knowledge and precise diagnosis stemming from such mastery requires an interdisciplinary view and clinical experience in the analysis of each case. 

Tooth resorptions, despite their multiple causes, are not multifactorial, since their manifestation does not require multiple conditions: one single cause is sufficient to promote it at the roots.^6^ Orthodontic movement constitutes just one of the causes of tooth resorption, albeit the most frequent. It is also the cause that promotes the least frequent tooth loss. 

The following two mechanisms can explain the emergence of root resorption: 

1) The death or removal of cementoblasts from the surface, when inflammation in the area will induce clasts to reabsorb the exposed mineralized dental tissue, giving rise to the process known as inflammatory tooth resorption.

2) The death of the epithelial remains of Malassez (ERM), which are responsible for the preservation of periodontal space^12^ via epidermal or epithelial growth factor (EGF) release. This event takes place only in the various modalities of dental trauma, but not in orthodontic movement, even in adverse conditions. The alveolar bone will encroach upon the periodontal space without ERM and the alveolodental ankylosis will include the tooth in the process of bone turnover. Thus, we will have replacement resorption that does not have as one of its causes orthodontic procedures.

Tooth resorptions in both mechanisms have local causes that remove cementoblasts and, sometimes ERM from the root surface. In the case of tooth resorptions associated with orthodontic movement, especially in the apical region, the cementoblasts are killed due to lack of oxygen caused by vessel compression during induced tooth movement.

Clinical practice is fraught with civil, ethical, and technical liabilities. Regardless of these liabilities, we cannot ascribe the causes of tooth resorptions to the organism, attributing to them susceptibilities and inherited factors to explain cases in which they occurred due to the incidence of applied forces. Tooth resorptions in clinical practice are acceptable as a consequence of orthodontic treatment, provided they are controlled and predictable.^6^ ^,^ ^28^

Tooth resorptions in orthodontic practice are not necessarily iatrogenic in nature, and must be considered as events inherent in orthodontic treatment, especially when taken into account in the planning and control of the patient. Iatrogeny related to tooth resorption is associated with misdiagnosis, practiced with either inappropriate methods or without assessment of its predictability.^6^ In orthodontic treatments in which planning and analysis of all teeth are conducted by means of periapical radiography or computerized tomography, and by taking into account predictive factors, tooth resorptions will not have an iatrogenic nature.

In orthodontic practice, tooth resorptions are predictable and can be prevented for the most part.^6^ ^,^ ^28^ Over time, many studies have attempted to explain or associate these resorptions with genetic, hereditary factors, individual and/or family susceptibilities and proclivities; systemic diseases such as endocrinopathies are also ascribed as a cause.^15^ ^,^ ^16^

In 2004, we published a series of six papers^7^ ^,^ ^8^ ^,^ ^9^ ^,^ ^10^ ^,^ ^11^ ^,^ ^13^ analyzing several studies on the non-existent relationship between tooth resorption and heredity, and investigating classic works^1^ ^,^ ^2^ ^,^ ^18^ ^,^ ^25^ on the subject. For each study, we presented its limitations and why they should not be considered as scientific foundations for establishing this relationship, which is not revealed in clinical practice. We finished this analysis in 2012, in the third edition of a book on dental resorptions in clinical specialties.^6^

In general, researchers worry excessively about the laboratory part of their studies and fail in the selection of the sample, in the standardization of technical and diagnostic orthodontic procedures, and especially in diagnosing, using failed methods such as orthopantomographic panoramic radiographs and cephalograms that do not serve the purpose of diagnosing alterations such as tooth resorptions.

Furthermore, but still approaching these works and interpreting their results, they also fail because they present misguided primary conceptual aspects of the basic sciences, in addition to limited sampling and diagnostic methodologies. From 2012 to 2016, several other works with the same limiting problems continued to be almost replicated in publications. In order to provide a simple yet effective collaboration to determine proper distinction and better understanding of why we should not consider resorptions as hereditary, we will now proceed to discuss the subject.

## IMPORTANT CONCEPTUAL ASPECTS USUALLY ADDRESSED IN THE WRONG WAY

The term genetic refers to genes, which are defined as comprehensive information recorded on the chromosomes of cell nuclei. Let us resort to an analogy to help in understanding this issue with propriety and depth, but always with simplicity.^6^ ^,^ ^13^

Genes can be compared to recipes compiled in a book; and this book would be the chromosome. Each human cell has 23 pairs of recipe books in their nucleus, composed of a material known as DNA. When some structure in the body requires the cell to perform a given function or produce something useful, a mediator is released to act on its surface receptors, or “biochemical ears”. Internally, this cell is modified to open one of the books, the chromosomes, on the page where the recipe (the information) is, thus biochemically revealing what should be performed. Once received, the order is then fulfilled.^6^ ^,^ ^13^

Before the activated gene can determine the function, a substrate must be made available to the cell. In proceeding with the cookbook analogy, to make the cake, one must have the ingredients available. In the cell, these ingredients would be enzymes, amino acids, peptides, proteins, proper pH, etc. A gene can be activated by many mediators; a function, or a recipe, may be required by many mediators. In other words, several mediators can request the cake to be baked. If one mediator does not provide the inducement, others will. Like in some cakes, some genes, or recipes, are very specific, but most phenomena can be induced by several mediators.^6^ ^,^ ^13^

Cellular functions are genetically managed, but this does not necessarily mean that their effect is hereditary or familial. Inheritance is related to the transmission of characteristics from parents to children. One example: the alteration that the solar rays induce in the cells and provokes cancer is genetic, but not hereditary. The white color of an individual, which is more susceptible to skin cancer is, in turn, hereditary. In short, not everything to which the genetic term is attributed is hereditary but, of course, for something to be hereditary, it must be genetic and present in the spermatozoa and ova.^6^ ^,^ ^13^

In order for tooth resorption to occur, the process should be started by removing the layer of cementoblasts from the root surface and exposing the mineralized part. If the neighboring cementoblasts do not replenish the lost layer, bone cells can be attracted and mobilized for root resorption. Numerous mediators are released into the periodontal ligament during orthodontic movement. These are cytokines, growth factors and arachidonic acid products, as well as inflammatory mediators, which are numerous and act simultaneously. The lack of one will be compensated by the whole set of events. Among these mediators we can mention interleukins, TNF, interferon, epidermal growth factor and TGFß, as well as prostaglandins, leukotrienes, etc. In order for cells to synthesize and release mediators they must read a gene from one of the chromosomes. This is therefore, a genetic event, but it does not impart a hereditary connotation to the effect.^4^ ^,^ ^6^ ^,^ ^9^ ^,^ ^13^

A greater or lesser production of one of these mediators in tooth movement is related to a greater stimulus in the periodontal cells, which is promoted by stress and inflammation as a function of the applied forces. To impart a hereditary connotation to tooth resorptions based on a genetic study of one of these mediators involved and diagnosed in maxillary incisors using cephalometric and panoramic radiographs may constitute a misconception and seriously undermine the credibility of the authors and journals that publish it. 

## FROM A METHODOLOGICAL POINT OF VIEW, ANY ATTEMPT AT CORRELATING ROOT RESORPTIONS IN ORTHODONTICS TO HEREDITY WILL INEVITABLY LEAD TO A LIMITED DIAGNOSIS

The key methodological failure committed by studies that seek to relate tooth resorption to a hereditary origin,^1^ ^,^ ^2^ ^,^ ^18^ ^,^ ^25^, despite being subtle, is in their means of diagnosis.^1^ ^,^ ^14^ ^,^ ^17^ ^,^ ^19^ ^-^ ^24^ ^,^ ^26^ ^,^ ^27^ ^,^ ^29^ ^,^ ^30^ In almost all of them, digitized panoramic orthopantomographs or cephalograms are used. These radiographs do not allow accurate diagnosis or measurements of apical tooth resorptions and undermine the credibility of the results despite biomolecular techniques to identify genes and mediators. 

To be accurate, tooth resorption diagnosis must be made from periapical radiographs and/or computed tomography. Orthopantomographs do not serve this purpose, even if digitized. Although not digital, panoramic radiographs afford richer detail for the diagnosis of dental alterations.

Another situation that is systematically repeated in these works concerns the number of patients. Considering the prevalence of the phenomenon, this number should be high enough as to allow conclusions to be drawn which are solid to the extent that one can safely attribute to heredity the phenomenon of tooth resorptions. In general, the works involve only a few dozen patients. In some studies, although the number of patients was reduced, no heredograms or other analyzes of family involvement were performed.

Standardization of the sample is almost always neglected. The types of planning and techniques are quite different from one study to each other, the number of evaluators is not standardized or limited, and various types of professionals are included, whose training and clinical background are diverse, ranging from students to professionals of various origins. The type of diagnosis and planning should be standardized, as should the evaluators. 

**Figure 1:**
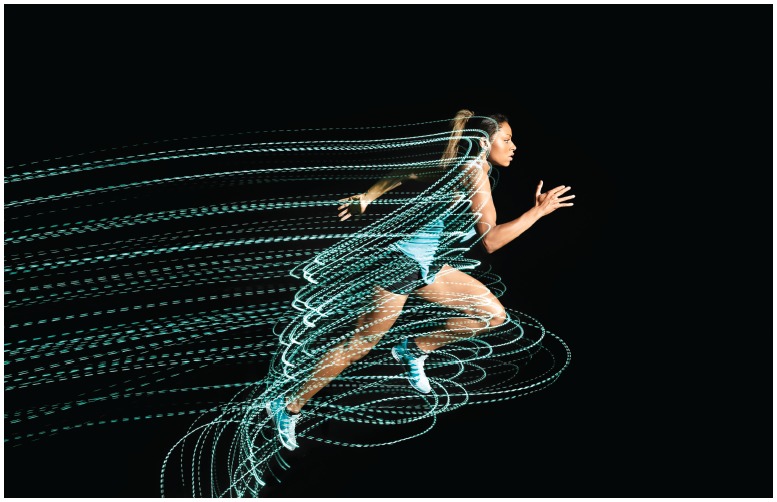
Induced tooth movement and tooth resorptions, like other biological events, occur with genetic information but this does not necessarily impart to them a hereditary nature. The terms “genetics” and/or “hereditary” are not synonymous!

This utter lack of standardization compromises almost totally the results, especially when the measurements of tooth resorptions are performed in orthopantomographs and cephalograms. It should be acknowledged that measuring millimeters of apical root loss using this type of dental image - often distorted - leads to very poor results even if, when analyzed statistically, it turns out significant. What makes these researches almost entirely useless is the manner in which the image is obtained, i.e., usually distorted, which cannot be evaluated by statistical methods.

## CONCLUDING REMARKS

So far, no consistent scientific evidence exists to substantiate or link in any way external inflammatory apical resorptions caused by orthodontic movement to hereditary factors, genetic or familial susceptibilities or proclivities. The studies addressing this issue are fraught with the same methodological and interpretive errors, especially those deriving from the diagnosis and measurement of root resorptions in orthopantomographs or cephalograms, as well as those from heterogeneous samples of clinical evaluators and planning types, or with reduced number of participants - taking into account the prevalence of tooth resorptions in the general population.

Almost all biological events in our body are encoded and managed from biochemical information in DNA, which are known as genes. The fact that certain activities, such as those of an inflammatory, cellular, and tissue-reabsorbing nature are performed by genes allows us to assign to it the adjective or property of genetic, but not necessarily hereditary. Genetic alterations will only be inherited when they are present in the germ cells of the species, that is, in the sperm and egg cells of the biological parents of the patients. In many phenomena, mediators and recipients, as well as their respective genes, can be modified by local, epigenetic factors, transiently or permanently, and for this reason, these genetic alterations will not be transmitted to the descendants of their bearers. 

In short, dental resorptions are not hereditary. Its phenomena are genetically executed like all biological events of the human body, i.e. having a genetic characteristic. However, this is not inherited or transmitted to other generations, which can be demonstrated via heredograms and other forms of familial studies.

In the present study, an attempt has been made to instigate in the readers a critical spirit when it comes to the relationship between heredity and tooth resorptions in orthodontics, especially by challenging the readers to discern the degree or percentage of contribution provided by following factors: heredity, the environment and unknown factors. 

Readers are hereby advised to always pose the following questions:

1. Would they not be trying to disguise as divine punishment or chance, represented here by inheritance, the cause of an expected and sometimes inevitable and predictable consequence of tooth movement?
2. Would they not be trying to hide an iatrogenic effect behind a biologically shallow clinical practice, and using heredity as an excuse or justification?

Ultimately, tooth resorptions in orthodontic practice are not necessarily iatrogenic in nature, since they must be considered as events inherent in orthodontic treatment, especially when they are taken into account in the planning and control of the patient. Iatrogeny and tooth resorptions are associated with misdiagnosis and practiced with equally equivocal methods, i.e., without assessing predictability.^6^ In orthodontic practice, when all teeth are analyzed and planned through periapical radiography or computerized tomography, considering the predictive factors, tooth resorptions will not be iatrogenic in nature and should be considered as one of the clinical complications likely to occur during treatment.
